# Research progress of traditional Chinese medicine as sensitizer in reversing chemoresistance of colorectal cancer

**DOI:** 10.3389/fonc.2023.1132141

**Published:** 2023-03-13

**Authors:** Xiang Lin, Xinyu Yang, Yushang Yang, Hangbin Zhang, Xuan Huang

**Affiliations:** ^1^ The First Clinical College, Zhejiang Chinese Medical University, Hangzhou, China; ^2^ Department of Gastroenterology, The First Affiliated Hospital of Zhejiang Chinese Medical University, Hangzhou, China

**Keywords:** traditional Chinese medicine, colorectal cancer, chemoresistance, sensitizer, chemotherapy

## Abstract

In recent years, the incidences and mortalities from colorectal cancer (CRC) have been increasing; therefore, there is an urgent need to discover newer drugs that enhance drug sensitivity and reverse drug tolerance in CRC treatment. With this view, the current study focuses on understanding the mechanism of CRC chemoresistance to the drug as well as exploring the potential of different traditional Chinese medicine (TCM) in restoring the sensitivity of CRC to chemotherapeutic drugs. Moreover, the mechanism involved in restoring sensitivity, such as by acting on the target of traditional chemical drugs, assisting drug activation, increasing intracellular accumulation of anticancer drugs, improving tumor microenvironment, relieving immunosuppression, and erasing reversible modification like methylation, have been thoroughly discussed. Furthermore, the effect of TCM along with anticancer drugs in reducing toxicity, increasing efficiency, mediating new ways of cell death, and effectively blocking the drug resistance mechanism has been studied. We aimed to explore the potential of TCM as a sensitizer of anti-CRC drugs for the development of a new natural, less-toxic, and highly effective sensitizer to CRC chemoresistance.

## 1 Introduction

As reported by Global Cancer 2020, colorectal cancer (CRC) ranks second and third in global cancer incidence and mortality (1, 2). It has become a great threat to human health. This high incidence rate can be attributed to the asymptomatic manifestations of early CRC and the lack of experience in endoscopic detection so that about 50% of all patients with CRC are at or above the local progressive stage at the time of their first diagnosis (3, 4). Reducing the incidence and mortality of digestive tract tumors and optimizing the treatment scheme of digestive tract tumors are some of the major public health issues that need to be addressed.

Presently, the treatment of CRC involves surgery, radiotherapy, immunotherapy, targeted therapy, and other comprehensive treatments (5). For patients with advanced CRC, chemotherapy is one of the most important treatments. Efforts have been made to use 5-fluorouracil (5-FU) combined with calcium folinate or oxaliplatin (OXA) as the first-line treatment for metastatic CRC, with a drug resistance rate was >40% (6). Some studies have even pointed out that 90% of patients with metastatic CRC died because of chemoresistance (7). This finding depicts that clinical drug resistance and chemoresistance are the major obstacles to the successful cure of cancer. Reducing chemoresistance, minimizing side effects, improving the patient’s prognosis, and quality of life are important factors to be taken into consideration during the development of new treatment options for advanced CRC.

Chemoresistance in CRC hampers the use of standard chemotherapy as a treatment option. Efforts have been made to develop treatment options relying on reversing chemoresistance by targeting newer cytotoxic drugs, regulating drug resistance to conventional drugs, and studying the changes of in tumor gene spectrum before and after chemotherapy. However, most of these strategies result in serious side effects, involve higher treatment costs, and face a crunch in technical expertise (8, 9). The benefits of natural products render them a promising alternative for treating cancer chemoresistance. It has been reported that 50% of the anti-cancer drugs approved by the Food Drug Administration (FDA) originate directly or indirectly from natural products or their derivatives (10). Traditional Chinese medicine (TCM) is multi-targeted and less toxic. TCM treatments are unique, safe, and effective for cancer therapy in China. It is also known to improve the quality of life, prevent metastasis and recurrence, resist chemoresistance, increase efficiency, reduce toxicity, and enhance immunity. Owing to these properties, TCM has gained tremendous research attention worldwide (11). Moreover, the synergistic effects of the TCM extracts and compounds (such as curcumin, berberine, *Sophora japonica*, and *Andrographis paniculate*) when combined with traditional chemotherapy could resist chemotherapy tolerance, which is noteworthy.

The present paper focuses on understanding the mechanism of chemoresistance of CRC and explores the potential of TCM and its active ingredients as chemosensitizers in reversing chemotherapy tolerance, reducing toxicity, and increasing the efficiency of CRC. Furthermore, the related mechanisms *in vivo* and *in vitro* have been summarized. Finally, based on the current research on the function mechanism of TCM, we believe that TCM as an anticancer treatment option seems promising in solving the difficulties of clinical anticancer treatment.

## 2 TCM reverses the chemoresistance of CRC and inhibits tumor growth *in vivo* and *in vitro*

### 2.1 Drug efflux

**Figure 1:**
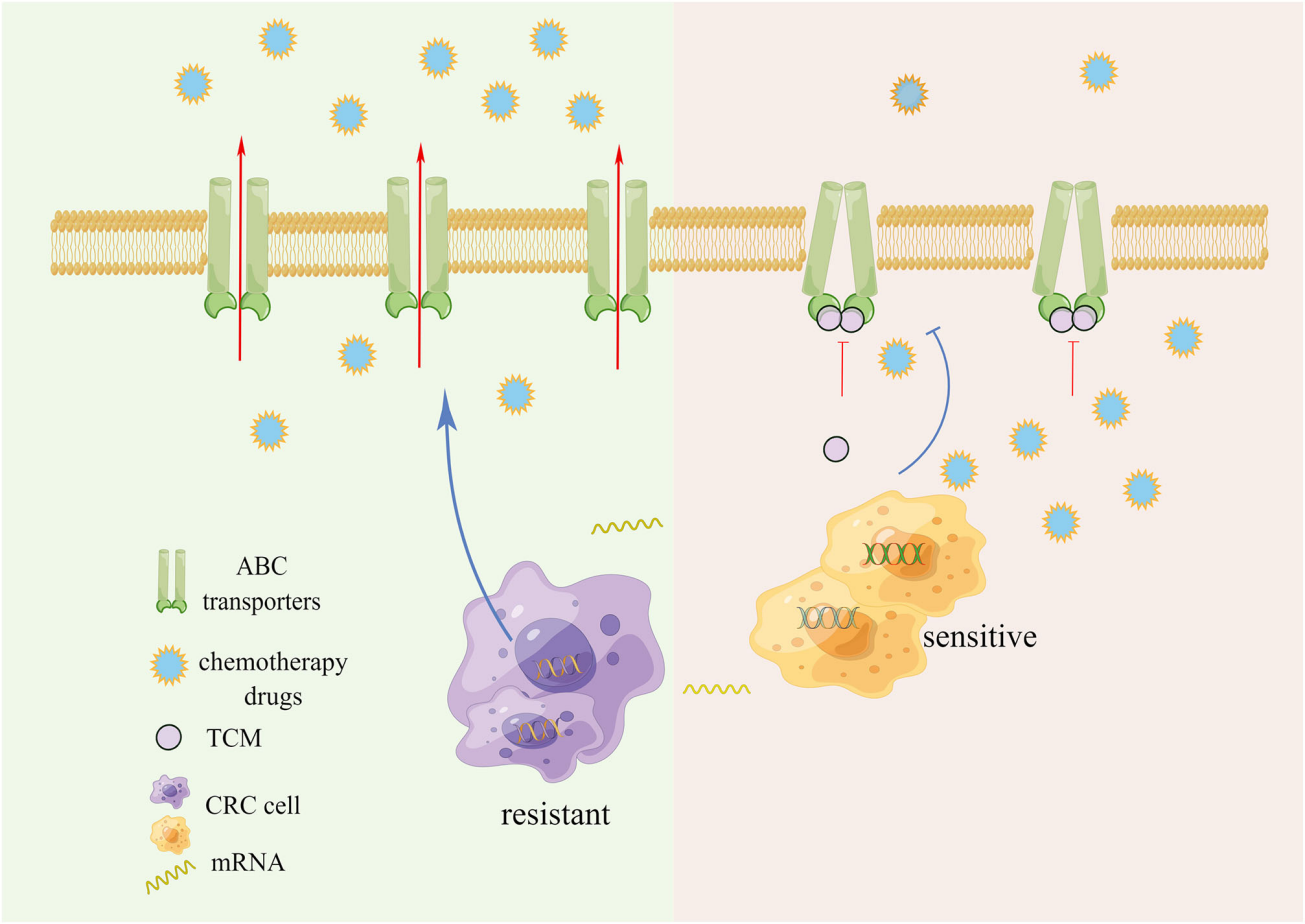
ABC transporters active drug efflux and chemotherapy resistance.

The chemical resistance of tumor cells is dependent on the outward transporter of the ATP-binding Cassette Family (ABC). Its increased expression often causes drug efflux, thereby leading to multiple drug resistance (MDR) (**Figure 1**). ABC transporter is a transmembrane protein that assists in transporting various substances across cell membranes. Upon substrate binding, ATP hydrolyzes and actively pumps drugs out of the cell, thereby preventing the accumulation of toxic substances within the cell. Similarly, in cancer cells, the efflux mechanism reduces the concentration of antitumor drugs within cancer cells, thereby protecting the cancer cells (12). It has been reported that the expression of ABC transporter is low in CRC cases, but significantly increased when drug resistance is induced in a variety of CRC cells (13–15).

Anti-MDR strategies include P- glycoprotein (P-gp) inhibitors, RNA interference, nano-drugs and combination drugs, but these transporter inhibitors have not yet shown definite clinical benefits (16–19). The importance of developing new inhibitors with high efficiency and low side effects is self-evident. Several studies have reported that a variety of TCMs and their active ingredients can effectively inhibit the expression and efflux of transporters, reverse the chemoresistance of CRC, and act as chemical sensitizers while exerting cytotoxic effects. Our previous research found that Gegen Qinlian Decoction can inhibit the expression of ABC transporters *in vivo* and *in vitro*, and synergize with Oxaliplatin to treat OXA-resistant CRC, and restore the sensitivity to OXA (20).

*Glycyrrhiza uralensis* Fisch. inhibited the efflux function of transporter P-gp in a CRC cell model and played a therapeutic role with *Aconitum carmichaelii* Debeaux, enhancing its efficacy and reducing toxicity (21). The ethanol extract of *Scutellaria barbata* D. Don enhanced the retention of rhodamine, the substrate of ABC transporter in 5-FU-tolerant CRC cells, leading to the inhibition of cell proliferation and cell apoptosis (22). Cucurbitacin E can inhibit TFAP4/Wnt/β-Catenin signaling, down-regulate the expression of ABCC1 and MDR 1 to increase the chemosensitivity of colorectal cancer cells to 5-FU (23). Quercetin and kaempferol regulated the activity and expression of MRP2, thereby promoting the absorption of drugs in the intestinal tract and inhibiting drug efflux as well as downregulating the expression of ABCB1 (24, 25). Neferine, isolated from lotus seeds, combines with P-gp, and increases the retention of drugs in cancer cells (26). Lignans, a secondary metabolite of *Arctium lappa* L., regulates the efflux function of P-gp in multidrug-resistant cancer cells (27). *Pien Tze Huang*, a TCM preparation, decreased the proportion of stem cell-like lateral groups of CRC cells in a dose-dependent manner, affecting the morphology of cancer cells, and the mRNA levels of ABCB1 and ABCG2 (28). Evodiamine inhibits the p50/p65 NF-κB pathway and inhibits ABCG2-mediated oxaliplatin resistance (29). On the basis of homologous protein modeling and molecular docking technology, 837 TCMs were reported to have the potential to inhibit the activity of ABCG2 (30). TCM and its active ingredients have demonstrated a good ability to inhibit ABC transporters, making them favorable candidates for developing new inhibitors.

### 2.2 DNA damage and repair

**Figure 2:**
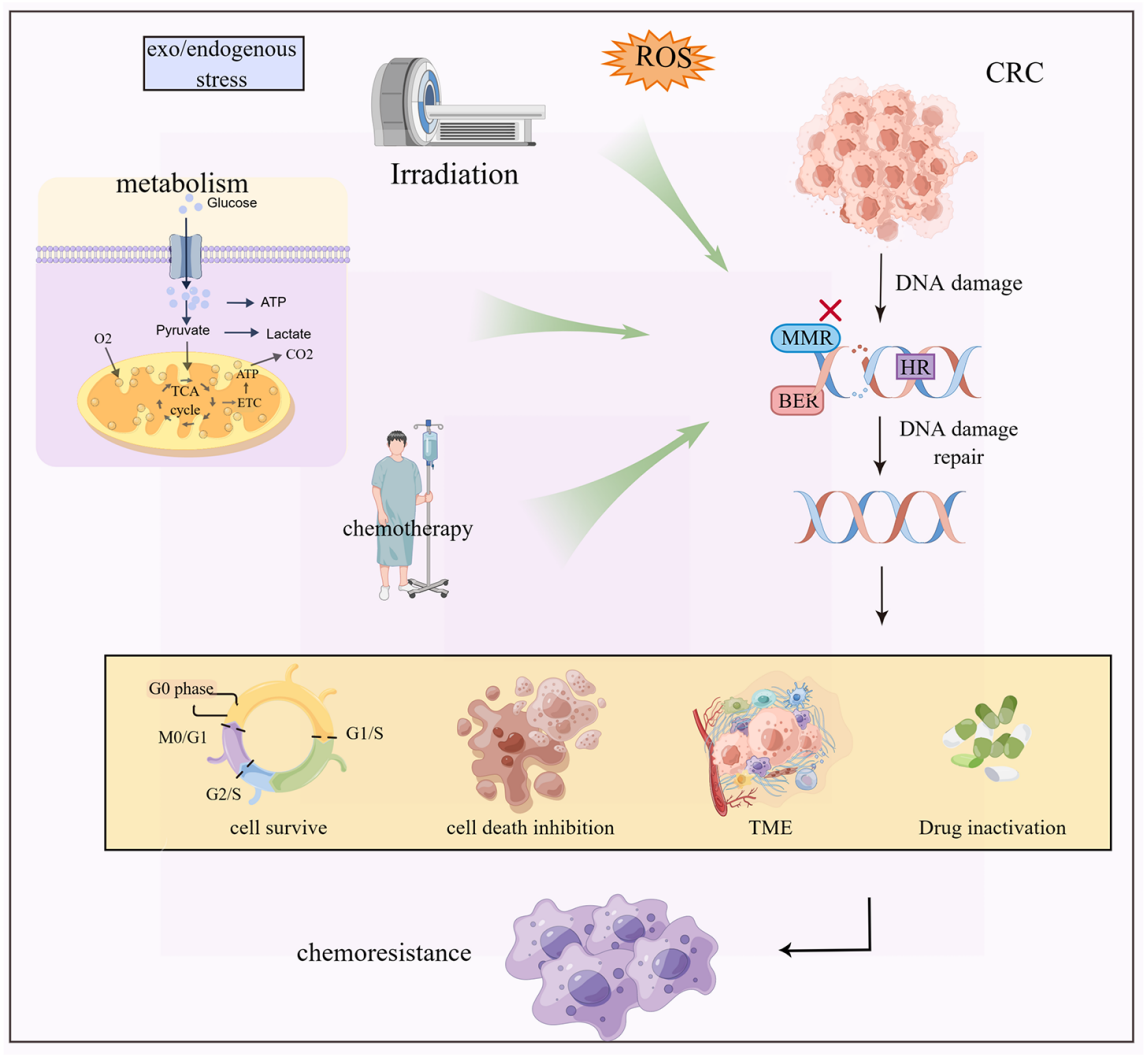
Exogenous and endogenous stresses such as radiotherapy and chemotherapy, metabolic reprogramming and ROS cause DNA damage and repair inhibition of CRC cells, which cause chemoresistance by regulating cell death, tumor microenvironment change and drug inactivation.

DNA damage repair mechanisms include base excision repair (BER), mismatch repair (MMR), and homologous recombination (HR). These mechanisms identify and correct DNA damage caused by multiple factors such as reactive oxygen species (ROS), radiation, and chemicals (31, 32). DNA damage repair, not only removes the damaged cells but also assists in drug resistance (**Figure 2**) (33, 34).

For example, 5-FU induces a base mismatch in DNA, which is recognized by MMR protein, leading to programmed cell death. Defective MMR prevents cancer cell death. BER recognizes and removes 5-FU in DNA causing drug resistance (7, 35, 36). In addition, nuclear DNA is the main target of platinum-cytotoxic drugs. These drugs covalently bind to DNA to form a Pt-DNA adduct, which damages the normal transcription and replication functions of DNA. This event activates a variety of signal pathways leading to cell apoptosis. Once the process of platinum damaging DNA is inhibited, the tendency of cell apoptosis will get weakened, resulting in drug resistance of the tumor (37–39). The challenges in anti-cancer platinum drugs are avoiding the precise and efficient DNA damage repair mechanisms, such as the HR repair mechanism of DNA double-strand breaks in cells. The mechanism of DNA damage combined with the inhibition of DNA repair results in sensitizing the cancer cells, thereby improving the therapeutic effect.

The use of TCM and prescriptions in DNA damage, the repair of cancer cells by reversing drug resistance, and alleviating the toxic side-effects of chemotherapeutic drugs are well documented (40–42). It has been reported that shikonin from the root of Lithospermum erythrorhizon enhances DNA damage caused by cisplatin by inducing intracellular oxidative stress and mitochondrial activation leading to the inhibition of HCT116 xenograft tumors in nude mice (43). Compound kushen injection led to the inhibition of cell cycle regulatory protein and DNA repair. It also promoted DNA double-strand break and induced cancer cell apoptosis through multiple pathways (44–46). Curcumin is known to regulate the expression of DNA repair-related genes, inhibit tumor growth, promote apoptosis, and enhance the chemosensitivity of MMR-deficient CRC to 5-FU (47–49). Berberine led to the downregulation of RRM1, RRM2, LIG1, and POLE2, participated in DNA repair and replication, and demonstrated the potential to inhibit DNA repair (50). Ginsenoside Rg3 responds to DNA damage by activating the VRK1/P53BP1 pathway, improving the efficiency of chemotherapy, reducing its side effects, and providing a new idea for cancer prevention and treatment (51). Cantharidin works by upregulating KDM4A and catalyzing the demethylation of histone H3K36me3 and inducing DNA damage, which in turn enhances the chemotherapy sensitivity (52). Cytotoxic drugs often result in serious neurotoxicity and other side effects while killing tumor cells, while Silibinin protects against cisplatin-induced neurotoxicity by reducing DNA damage and apoptosis (42, 53, 54).

### 2.3 Cell death

**Figure 3:**
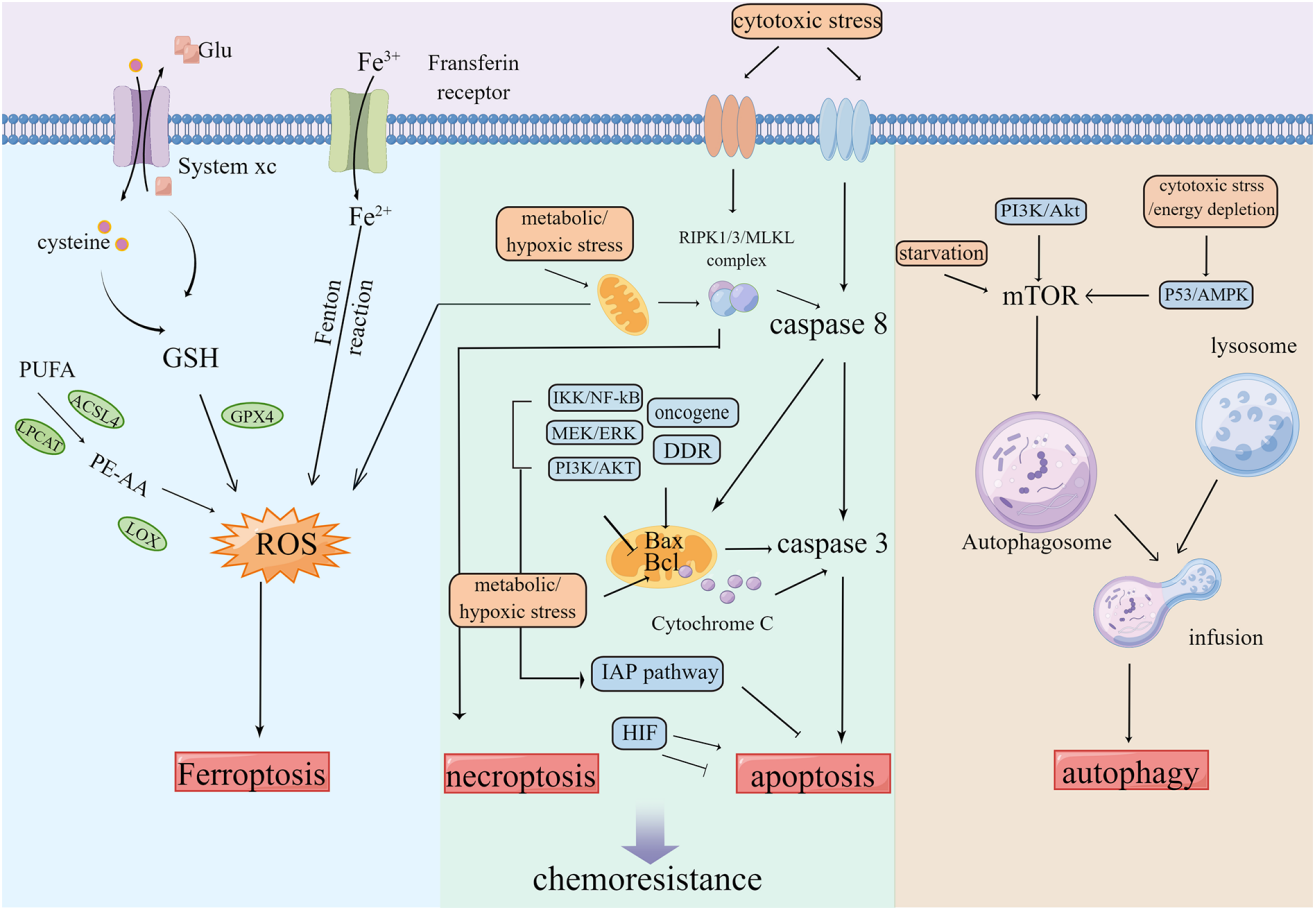
Tumors keep their sustained growth, metastasis and gain chemoresistance by escaping cell death such as ferroptosis, apoptosis and autophagy etc.

The programmed cell death pathway is necessary to maintain normal cellular development, which includes apoptosis, necrosis, cell scorch, iron death, and cell death modes related to autophagy and non-programmed necrosis (55, 56). Several studies have validated that apoptosis, autophagy, and other cell death modes are closely related to chemotherapy resistance (**Figure 3**). Multiple factors such as hunger and chemotherapy cause stress to the cancer cells. The uncontrolled growth of tumor cells leads to increased metabolic demand, which then activates autophagy, leading to the inhibition of tumors by the removal of damaged proteins/organelles (57). The rapid proliferation of cancer cells increases the nutrition and oxygen demand. Throughout treatment, cancer cells are in hypoxia and nutritionally deficient environments. For survival, the cancer cells are significantly dependent on autophagosomes for metabolism. Thus, autophagy fails to inhibit tumor growth, but instead results in chemotherapy resistance, thereby contributing to the development of MDR and protecting cancer cells from the attack of chemotherapy drugs (58–60).

Orderly death is called apoptosis, which is essential in maintaining the internal cell environment. Tumor cells lack the ability of apoptosis (20, 61). Tumor cells adapt to the induction of apoptosis by drugs, which leads to the development of drug resistance. An effective way to overcome drug resistance related to apoptosis of cancer cells is through stimulating and restoring the ability of tumor cells to undergo apoptosis and promoting the death mode independent of apoptosis (62–64).

Ferroptosis is a type of event that involves the accumulation of lipid-ROS (L-ROS) in an iron-dependent manner (65, 66). Glutathione (GSH) depletion inactivates GSH-dependent peroxidase (GPX4). Transferrin containing Fe^3+^ enters the cell and transforms into Fe2+ to participate in the Fenton reaction. The unsaturated fatty acids, such as arachidonic acid, are metabolized into PE-AA through ACSL4 and LPCAT, which are then oxidized by LOXs, all of which can lead to the accumulation of L-ROS and further ferroptosis of cells (67, 68). The inhibition of ferroptosis can promote the development of tumors, resulting in resistance to several types of chemotherapy (69–71).

Reversing the inhibited cell death mode, developing new cell death modes, and inducing cell death are important means to overcome chemoresistance. Various compounds from TCM have been proven to target a variety of cell death modes and reverse the drug resistance induced by chemotherapy drugs. For instance, the combination of artesunate and autophagy inhibition blocked the protective effect of artesunate-induced autophagy on CRC cells and enhanced the apoptosis induced by artesunate (72). *Zuojinwan* induced apoptosis of HCT116 cells by the PI3K-Akt-signaling pathway (73). Curcumin upregulates CDX2, thereby inhibiting the Wnt/β-catenin signaling pathway and downregulating the anti-apoptosis signaling. This event ultimately results in reduced cell viability and apoptosis of human colon adenocarcinoma cell SW620 (74–77). Pien Tze Huang inhibits STAT3 and the Notch1 pathways and promotes apoptosis of CRC cells (78, 79). *Sanguisorba officinalis L.* inhibits the Wnt/β-catenin pathway, downregulates Bcl-2 protein, upregulates pro-apoptotic proteins such as Bax, and inhibits the growth and metastasis of 5-FU-resistant CRC (80). Moreover, cisplatin is a known apoptosis inducer. The combined use of the compound kushen injection and cisplatin enhances cell death of SW480 by inducing exogenous apoptosis (46, 68).

Several studies have reported that Chinese medicinal compounds and their active ingredients are involved in the induction and inhibition of autophagy of cancer cells. Autophagy works by inhibiting tumor growth through the removal of damaged organelles and the induction of autophagy under appropriate conditions. *Reynoutria japonica* Houtt. extracts induce autophagy and promote the apoptosis of cisplatin-resistant cancer cells (81). *A. carmichaelii* Debeaux induces apoptosis and autophagy, thereby reversing multidrug resistance (82). The treatment of CRC cells with T33, a compound of Chinese medicine, caused changes in autophagy markers, the activation of autophagy, inhibition of tumor proliferation, and reduction in the transplanted tumor volume (83). At the same time, autophagy is also a protective response to DNA damage after drug treatment (84, 85). The increase in the expression of the autophagy gene enhances the vitality of cancer cells and prevents cancer cells from entering apoptosis (72, 86). Moreover, it exhibits a protective effect on cells, resulting in autophagy-dependent chemotherapy resistance (60). It has been reported that resveratrol and epigallocatechin gallate (EGCG), the active ingredient of green tea, enhanced cell death by blocking the drug-induced protective autophagy flux and demonstrated synergistic anticancer effect, thereby overcoming drug resistance (87–89). Flavonoids from *S. baicalensis* Georgi inhibited autophagy, promoted cell apoptosis, and exerted anti-CRC activity by regulating the ATF4/sestrin2 pathway (90). *Sophora flavescens* Aiton extract activated the AMPK/mTOR pathway, increased the autophagy flux of colon cancer, and induced apoptosis (91).

Ferroptosis-assisted reverse chemotherapy resistance can be achieved by regulating the GPX4 pathway, iron metabolism pathway, and lipid metabolism pathway (69, 92). Genome analysis revealed that HMOX1, TP53, and ACSL5 were significantly upregulated in activated ferroptosis. This phenomenon was recorded in *Andrographis paniculata*-mediated CRC chemical sensitivity to 5-FU (93). *Camellia nitidissima* C. W. Chi decreased the expression of GPX4 and increased the expression of HMOX1 *in vivo* and *in vitro*. It also induced ferroptosis to inhibit CRC progress (94). β-elemene, a zedoary turmeric extract, induced ferroptosis through iron-dependent ROS accumulation, GSH consumption, and upregulation of transferrin. The downregulation of negative regulatory proteins of ferroptosis such as GPX4 and GLS, when combined with cetuximab, sensitized KRAS mutant CRC cells (95). Auriculasin isolated from *Flemingia philippinensis* promoted ROS production, accumulated intracellular Fe^2+^, and induced CRC ferroptosis in a concentration-dependent manner (96). MAP30 isolated from *Momordica charantia* seeds along with cisplatin stimulated cytoplasmic oxidative stress and ROS accumulation, induced ferroptosis, and mediated cancer cell death by increasing intracellular Ca^2+^, thereby exhibiting effective anticancer and chemoresistance effects (97). Glycyrrhetinic acid nanoparticles increased the ROS level in CRC cells by downregulating GPX4 and enhancing Fe-dependent cytotoxicity to kill CRC cells by Fenton reaction (98).

### 2.4 Drug inactivation and target changes

**Figure 4:**
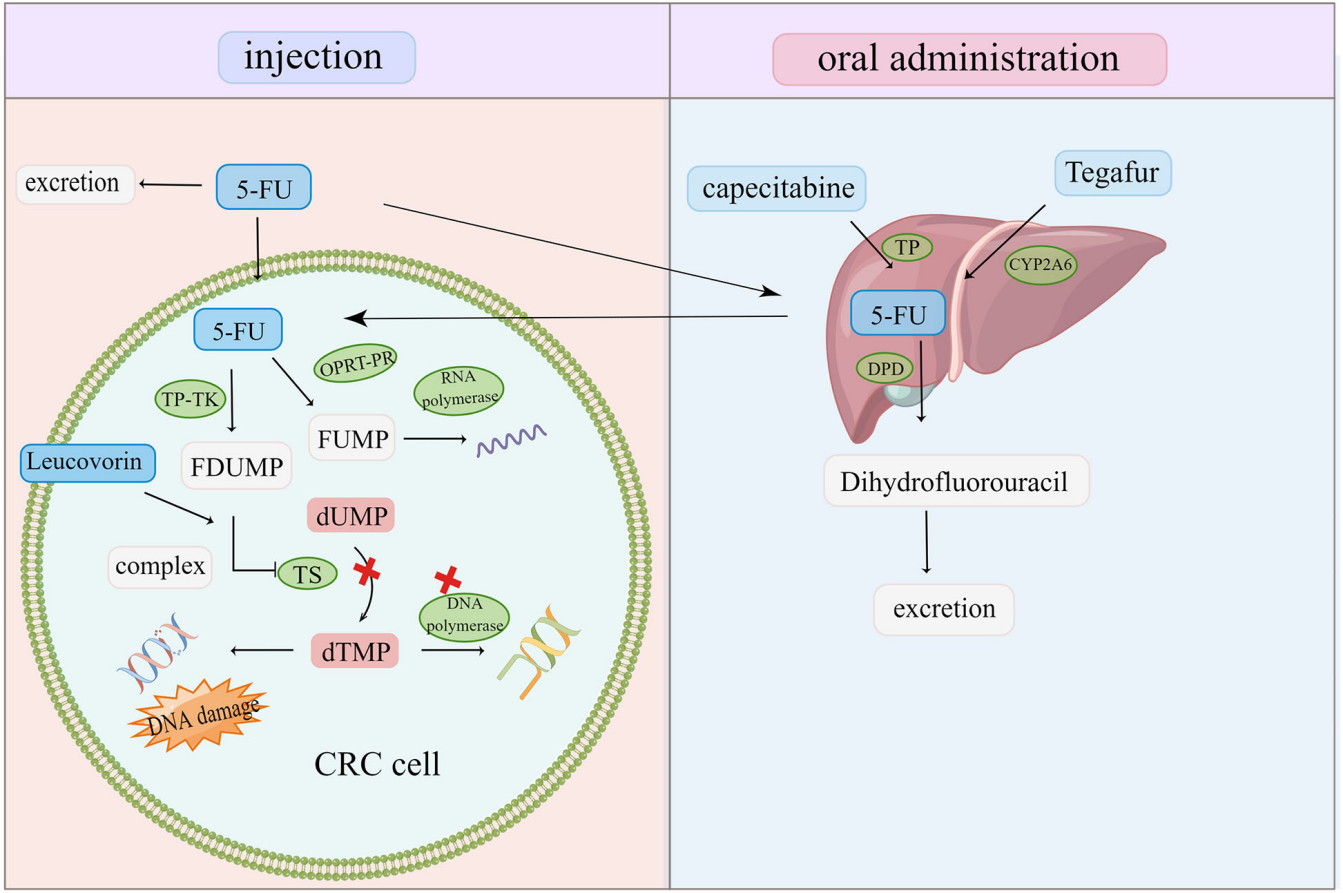
Oral and injected chemotherapeutic drugs are transformed into cytotoxic compounds after being activated and metabolized by various enzymes in the body. Once the target is changed and the drug is inactivated, CRC cells escape and become resistant to chemotherapy drugs.

5-FU is the cornerstone of first-line CRC therapy. However, clinical resistance to 5-FU and its derivatives remains a challenge in CRC treatment (36) (**Figure 4**). *In vivo*, 5-FU is transformed into FU deoxynucleotide (F-duMP) *via* the OPRT-RR and TP-TK pathways, inhibiting thymidylate synthase (TS), preventing dUMP methylation to dTMP, inhibiting DNA synthesis, and arresting the cell cycle in the S phase (7). Several studies have reported that gene polymorphism and TS upregulation in CRC are closely related to 5-FU tolerance (7, 99, 100). Thymidine phosphorylase (TP) is upregulated in CRC, which has a poor prognosis. Capecitabine is one of the most active oral fluoropyrimidines. Capecitabine has no cytotoxicity as a precursor of 5-FU, and it is activated by TP to become cytotoxic 5-FU; hence, its adverse reactions are mild and manageable (101). Increasing TP expression is required for chemotherapy drugs such as capecitabine to exert an anti-tumor effect. However, TP has significant angiogenic activities and anti-apoptosis properties, which promote tumor growth and metastasis (102, 103). A TP inhibitor eliminates the tumorigenic effect of TP and the activation of capecitabine. The dual nature of TP also affects chemical resistance. TAS-102 is an oral FU preparation composed of trifluoropyridine (TFT) and thymidine phosphorylation inhibitor (TPI) (104). TFT can inhibit TS from hindering DNA synthesis, but it can also induce DNA damage and demonstrate a cytotoxic effect by doping its metabolites into DNA. In addition, a TP inhibitor prevents TFT degradation by TP and ensures the TFT biological activity (105). This unique action mechanism makes it suitable for patients with CRC with 5-FU and OXA resistance, hence it can be employed in the clinical treatment of metastatic CRC (106).

5-FU is excreted before getting metabolized to dihydrofluorouracil by dihydropyrimidine dehydrogenase (DPD). A high DPD activity makes 5-FU inactive and tolerant (107, 108). Past studies have demonstrated that using DPD inhibitors to downregulate DPD in CRC and increase 5-FU bioavailability can effectively reverse 5-FU resistance (109). However, the toxicity of 5-FU caused by low DPD activity renders chemotherapy unsustainable (110).

The CYP450 enzyme mediates the inactivation of several anticancer drugs and the activation of precursor drugs. The overexpression of CYP450 subtypes such as CYP3A4 and CYP3A5 in tumor cells renders chemotherapy drugs, such as irinotecan, ineffective, resulting in chemical resistance (111, 112). CYP1A2 and CYP2A6 were significantly upregulated in 5-FU-resistant CRC cells, while the combination of CYP450 inhibitors restored 5-FU cytotoxicity (113).

GSH-transferase (GST) catalyzes the combination of drugs with GSH and excretes it *via* bile/urine, causing it to detoxify and inactivate, thereby reducing the efficacy of chemotherapy drugs (7). GST overexpression and gene polymorphism are linked to cancer progression and resistance to chemotherapeutic drugs such as OXA (114, 115). Its inhibitor should therefore be administered to boost chemotherapy’s curative effect and alleviate drug resistance.

The overexpression of TS desensitizes CRC to 5-FU drugs, and the overexpression of drug metabolic enzymes such as the CYP450 enzyme inactivates chemotherapeutic drug metabolism, allowing tumor cells to escape. Inhibiting drug target upregulation and lowering drug metabolic inactivation are essential approaches to increase chemotherapeutic sensitivity. Anti-tumor active ingredients derived from traditional Chinese medicinal plants and animals are gaining popularity. By inhibiting TS, formula HQGGT enhanced the toxic effect of 5-FU on 5-FU-resistant CRC cells and decreased tumor growth *in vivo* (116). *Venenum bufonis* extract degrades TS by the proteasome to limit TS expression, induce DNA damage, and hinder tumor cell proliferation (117). The expression of drug resistance-related genes TS and DPD in CRC cells was significantly downregulated after treatment with the formula GCFF, and 5-FU cooperated with the inhibition of cancer cell proliferation (118). *Coptidis rhizoma* extract can be utilized as an adjuvant to assist 5-FU in significantly inhibiting TS activity overexpressed in HCT116/R cells (119). Apigenin inhibited the TS expression and activity in 5-FU-resistant HCT116 cells as well as improved the 5-FU’s therapeutic efficacy on CRC (100). Emodin exists in a variety of herbs. It has been reported that emodin may raise the expression of TP mRNA and protein in cancer cells treated with capecitabine, enhancing capecitabine activation, and downregulating Rad51 and ERCC1, as well as cooperating with capecitabine-induced cytotoxicity (120). *S. baicalensis Georgi, S. flavescens*, and *Schisandra chinensis* (Turcz.) Baill. extracts significantly inhibited cytochrome P450 subtype activity in human liver microsomes (121–124). *Curcumae rhizoma* and *Arisaematis Rhizoma Preparatum* are two Chinese medicinal plants that have considerate inhibitory effects on CYP3A4 (125).

### 2.5 Epithelial-mesenchymal transition

**Figure 5:**
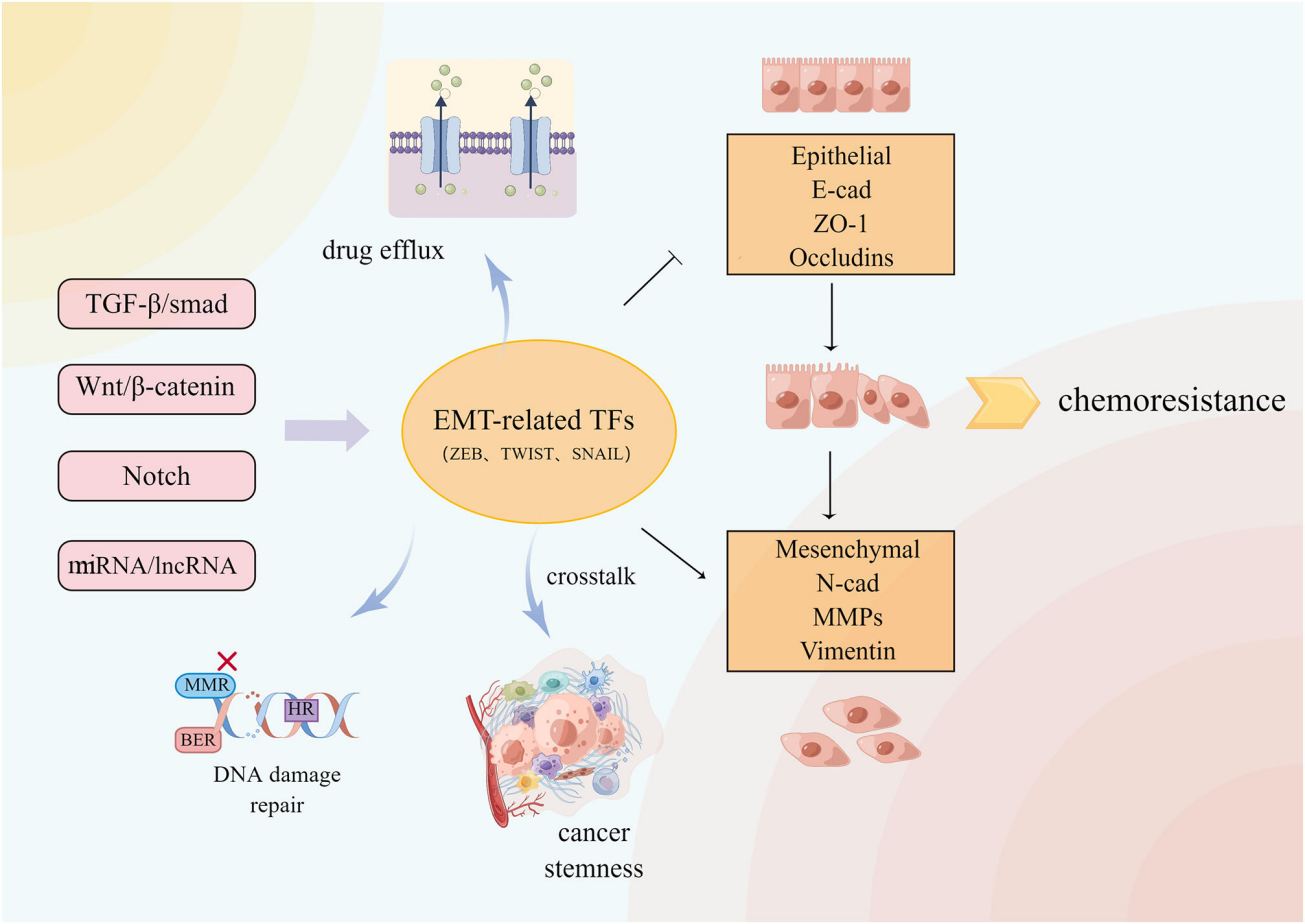
A variety of signal pathways regulate EMT-related transcription factors and downstream related pathways. EMT and crosstalk with TME, DNA damage repair and so on cause CRC resistance.

Epithelial-mesenchymal transition (EMT) is a reversible process in which polar epithelial cells lose the adhesion polarity of the basement membrane and the ability of the tight junction and cell adhesion due to the action of some factors, transforming the cells into mesenchymal cells that are capable of infiltration and migration (126). EMT in several physiological and pathological processes including wound healing, nerve development, and cancer cell metastasis. EMT is involved in several signaling pathways, which include the Notch, Wnt, and TGF-β signaling pathways (127).The changes in epithelial gene markers like E-cadherin and ZO-1 as well as mesenchymal gene markers such as N-cadherin indicate the occurrence of EMT. In recent years, the relationship between EMT and MDR has grown stronger (**Figure 5**). It was found that silencing the E-cadherin expression altered the chemoresistance of various CRC cell lines to irinotecan and oxaliplatin (128). EMT induced by the hERG1 ion channel reduces cancer cell sensitivity to cisplatin (129). BMAL1 knockdown increases the expression of epithelial markers in CRC cell lines while decreasing the expression of mesenchymal markers, maintains the balance between epithelium and mesenchymal, stabilizes cancer cells in the epithelial characteristic state, inhibits EMT, and reduces the chemical resistance of colon cancer cells (130). Inhibiting the Wnt signaling pathway delays the progress of EMT and reverses CRC chemical resistance to 5-FU (41). The EMT-induced protein ZEB2 promotes oxaliplatin resistance in CRC by activating the nucleotide excision repair genes *ERCC1* and *ERCC4* (131).

Cancer chemoresistance is frequently accompanied by EMT, which generates chemical resistance either directly or indirectly, although the mechanism by which EMT drives chemoresistance remains elusive. EMT-mediated chemoresistance is closely related to ABC transporter, tumor microenvironment, and cancer stem cells (CSC). The ABC transporter gene promoter contains the binding sites for EMT transcription factors such as Snail and ZEB. The overexpression of these sites increases the transporter promoter activity in cancer cells, resulting in transporter overexpression and drug efflux (132). EMT cells contain stem cell-like characteristics similar to CSCs in signal pathways and chemoresistance phenotype (133). The FBXW7-ZEB2 axis connects EMT with the tumor microenvironment to promote stem cells and CRC chemoresistance (134). Cancer-associated fibroblasts (CAFs) in the tumor microenvironment can activate Wnt/β-catenin and promote CRC stemness and 5-FU/L-OHP resistance by secreting exosome miR-92a-3p and transferring it into CRC cells (135). Although it is unclear how EMT contributes to chemoresistance, it is evident that inhibiting EMT is an effective measure to reverse chemoresistance and inhibit tumor growth and metastasis.

TCM and its extracts have had a robust response in this regard. Curcumin can reverse 5-FU resistance in CRC cells by regulating the TET1-NKD-Wnt signaling pathway and inhibiting EMT progression, as well as inhibiting the TGF-β/Smad2/3 signaling pathway and reversing OXA resistance in CRC (136, 137). Curcumin can also sensitize 5-FU-resistant cells by upregulating EMT inhibitory miRNA and downregulating BMI1 and other *EMT* genes (138). Cinnamaldehyde, derived from *Cinnamomum cassia*, can inhibit the Wnt/β-catenin pathway induced by hypoxia in conjunction with oxaliplatin, reverse EMT and CRC cells dryness, and improve CRC sensitivity to OXA (139). *S. officinalis L.* blocked the Wnt pathway, which inhibited the proliferation and metastasis of 5-FU-resistant CRC by downregulating N-cadherin, vimentin, and snail protein and upregulating E-cadherin (80). Danshensu inhibits platelet-induced EMT transformation and chemoresistance in CRC cells (140). Jiedu Sangen Decoction inhibits the AKT/GSK-3β signaling pathway, thereby reversing EMT (141, 142). Resveratrol strongly inhibited the formation of EMT and CSC induced by TNF-β, downregulated cancer-promoting factors such as vimentin and slug, upregulated E-cadherin and claudin-2, increased the tight junction and cell adhesion, inhibited NF-κB activation, and increased 5-FU sensitivity in 5-FU-resistant CRC (143, 144). Resveratrol can also inhibit the EMT progression in LoVo cells *via* the TGF-β1/Smads pathway (145). Quercetin inhibits TGF-β1-induced EMT by inhibiting Twist1 and regulating E-cadherin (146). β-element combined with cetuximab promoted the epithelial marker expression, decreased mesenchymal gene marker expression, inhibited EMT, re-sensitized KRAS mutant CRC cells against EGFR antibody therapy, and inhibited cancer cell migration (95). By promoting the miR-134 expression, astragaloside IV could downregulate the CREB1 signaling pathway, inhibit EMT, and increase the sensitivity of CRC to OXA (147).

### 2.6 Tumor microenvironment

**Figure 6:**
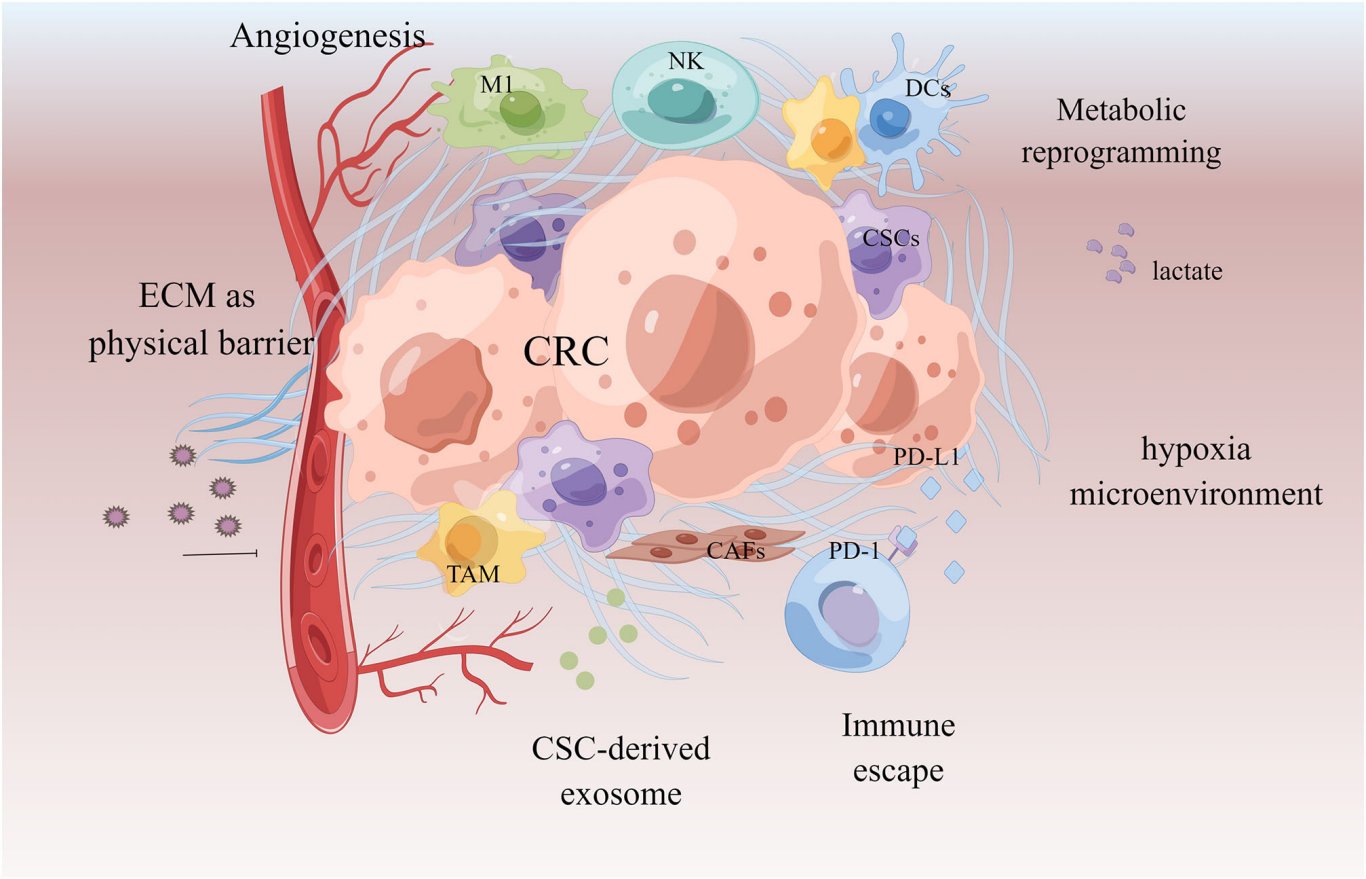
Tumor microenvironment in chemoresistant CRC. Extracellular matrix acts as a physical barrier to prevent drugs from entering. The microenvironment of the formation of new blood vessels, hypoxia and acidification contributes to chemoresistance. Cancer-associated fibroblasts (CAFs) and cancer stem cells (CSCs) promote tumor by secreting tumor growth factors and exosomes. Immune cells acquire immunosuppressive phenotype to make the tumor escape.

The mechanism of the tumor microenvironment (TME) in the MDR phenotype is becoming clearer. TME is an extremely complex ecosystem composed of tumor-associated macrophages (TAM), immune cells, cancer-associated fibroblasts (CAFs), and other non-cellular components such as secretory products and extracellular matrix (148). The cells and molecules in the tumor microenvironment can be exploited to maintain tumor immunosuppression and to promote immune escape, invasion, and treatment resistance. Cell components enhance tumor drug resistance by recruiting and secreting a variety of protective cytokines; non-cellular components, such as extracellular matrix (ECM) and hypoxia, can mediate drug resistance by building physical barriers and affecting tumor cell growth and metabolism (149–151). TAM secretes IL-6 and MFG-E8, respectively, activates the IL-6R/STAT3 and Hedgehog pathways, induces CSCs, and shields CSCs from the cytotoxic effects of 5-FU and cisplatin, resulting in treatment resistance and recurrence (152–154). CAFs transfer exosomes miR-92a-3p and lncRNA H19 into CRC cells, activate Wnt/β-catenin, mediate EMT, and promote CRC stemness and 5-FU/OXA resistance (135, 155). The hypoxic HOTAIR/miR-1277-5p/ZEB1 axis mediated oxaliplatin resistance in CRC (156, 157). Hypoxia can also interact with other mechanisms, such as activating cytochrome P450 subtype CYP3A6 and accelerating drug metabolism; it can also induce ABC transporter upregulation and promote drug efflux (158). Drugs’ effects are opposed and limited due to the intricacy of the tumor microenvironment. The therapeutic scheme targeting tumor microenvironment is becoming one of the most promising anti-cancer strategies in the future as our understanding of it grows (**Figure 6**).

TCM has been proven in studies to regulate TME-related molecular mechanisms and serve an anti-tumor role (157). Curcumin can enhance the FOLFOX’s cytotoxic effect on colon cancer by regulating EGFR and IGF-1R (159). Formula PHY906 can increase CPT-11’s anti-tumor activity by reducing neutrophil or macrophage infiltration, TNF-α expression, and pro-inflammatory cytokine concentrations, inhibiting NF-κB and lowering irinotecan (CPT-11)’s gastrointestinal toxicity (160). Gegen Qinlian Decoction combined with PD-1 inhibitor could significantly increase CD8+T cells in the peripheral blood and tumor tissues, reshaped the tumor microenvironment, inhibited immune checkpoint, and effectively restored the T cell functions (161). Ginsenosides can regulate the quantity and function of bone marrow immunosuppressive agents, enhance the body’s anti-cancer ability, and inhibit cancer cell growth, metastasis, and recurrence in a tumor microenvironment (162). When CRC cells are in the hypoxia microenvironment, tanshinone IIA inhibits angiogenesis by targeting TGF-β1 and inhibiting the HIF-1α/β-Catenin/VEGF pathway (163). Resveratrol inhibits tumor stem cell-like phenotype induced by TNF-β and increases chemotherapy sensitivity (144). Pien Tze Huang inhibited the Notch1 pathway *in vivo* to negatively regulate CSCs characteristics, prevent spheroid formation, and diminish tumorigenicity (78). The water extract of Huaier can inhibit the proliferation of CRC stem cells by downregulating the Wnt/β-catenin pathway (164). Berberine, an immunological checkpoint inhibitor, can enhance the immunity of tumor-infiltrating T cells, activate regulatory T cells, and diminish immunosuppressive myeloid-derived suppressor cells (MDSC) (165). Andrographolide, in combination with a PD-1 inhibitor, improved the function of CD4 and CD8 T cells, affected the expression of T cell-related markers, and inhibited the growth of CRC (166). Gallic acid combined with anti-PD-1 antibody can block PD-L1/PD-1 signal transduction, downregulate Foxp3 stability, inhibit regulatory T cells, promote CD8 T cells to secrete IFN-γ, and limit CRC (167). Nano-drugs containing ursolic acid and lentinan can destroy immunogenic cells, promote dendritic cell maturation, repolarize TAM into anti-tumor M1 type, reshape immunosuppressive TME, and render it sensitive to immunotherapy (168).

### 2.7 Epigenetic modifications

**Figure 7:**
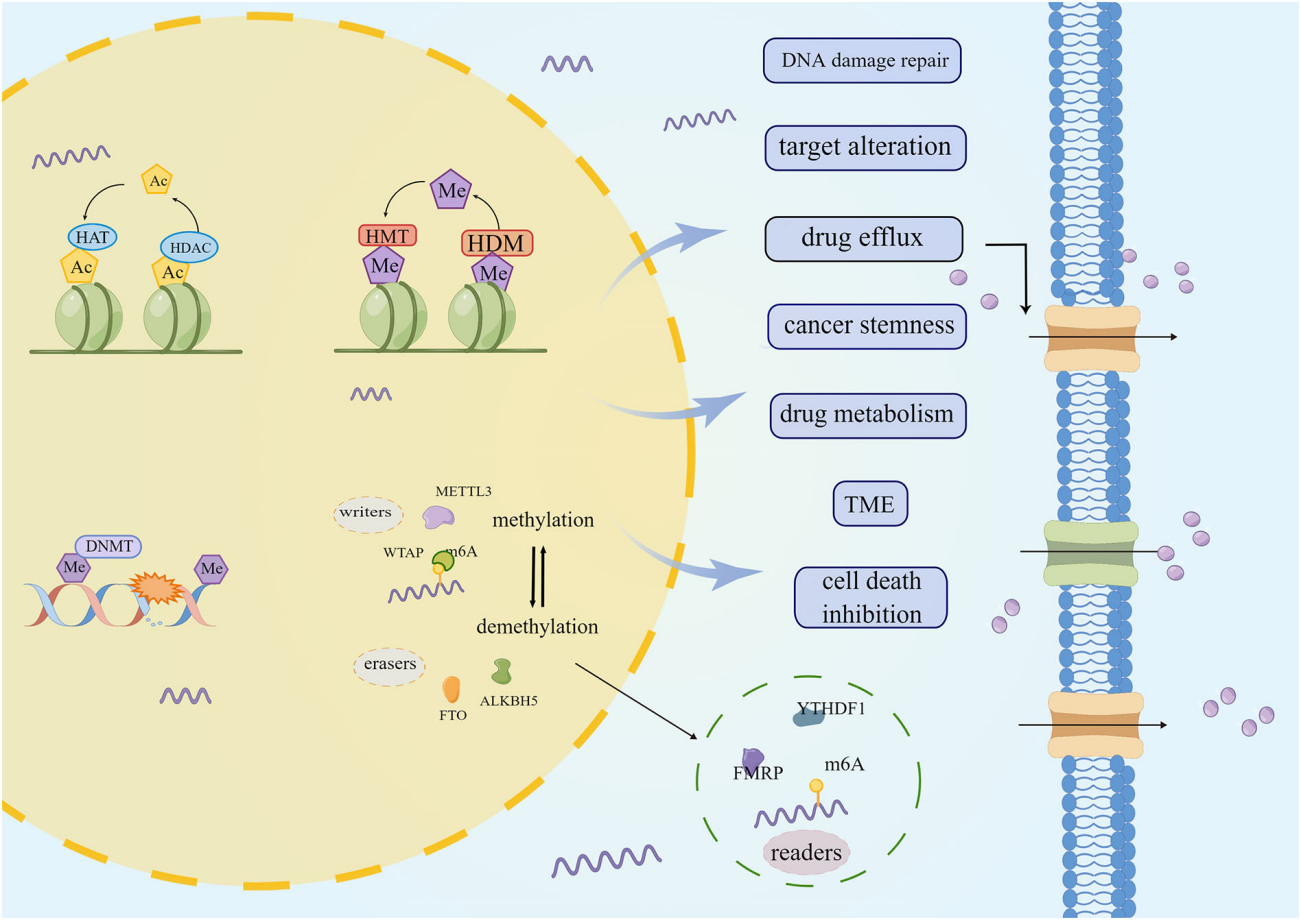
The role of epigenetic modification in chemoresistant CRC. DNA methylation, RNA methylation, histone methylation, deacetylation, etc., lead to CRC chemoesistance by promoting drug efflux, changing drug metabolism and cell death and other ways.

Despite the continuous development of treatment modalities, chemoresistance remains the most significant barrier to the curative treatment of various cancers, while epigenetics modification also plays a key role in the occurrence and development of chemotherapy resistance in tumors (**Figure 7**). Epigenetic modification influences cancer cell proliferation, metabolism, and metastasis, leading to anti-cancer drug resistance (169, 170). Histone alteration, such as methylation and deacetylation, interferes with the efficacy of drugs by regulating the expression of multidrug efflux transporters, drug-metabolizing enzymes, and drug targets (171–178). In addition, by regulating DNA damage repair, cancer stem cells, and tumor microenvironment, epigenetic modification protects cells against drug-mediated cell death (179–181). Currently, the emergence of several epigenetic inhibitors, such as DNA methyltransferase inhibitors and histone methyltransferase inhibitors, may make epigenetics a viable treatment strategy for reversing CRC drug resistance. When compared to traditional chemotherapy drugs, epigenetics drugs offer certain hope, although several problems still need to be addressed. DNMT inhibitors azacytidine and decitabine can cause bone marrow suppression and trigger gastrointestinal reactions (182). The performance of natural compounds in inhibiting epigenetic changes attracts people’s attention.

Curcumin has several epigenetic inhibitory activities. *In vivo* and *in vitro*, it can suppress the activity and expression of histone deacetylases in CRC, and reactivate the tumor suppressor gene *RARβ* (183, 184). Curcumin can be utilized to inhibit DNA methyltransferase, demethylate DNA, reverse decitabine tolerance in CRC (185). The combination of emodin and decitabine can significantly increase DNMT1 and DNMT3a expressions, improve the demethylation of tumor suppressor genes *P16*, *RASSF1A*, and *ppENK*, and play an anti-cancer role (186). Cinnamic acid derivatives inhibit histone deacetylase, which causes CRC cell death (187). Berberine lowered m6A *via* inhibiting β-catenin and increased FTO expression, which suppressed the stemness and tumorigenicity of CSCs in CRC (188). BBR binds to CSN5, blocking its ubiquitination activity, causing PD-L1 to be ubiquitinated and degraded, and preventing cancer cells from passing through immune checkpoints (165). Aidi injection inhibits ubiquitin-proteasome system activity, diminishes cytotoxic protein degradation, and targets mitochondrial apoptosis (189). TCM can target epigenetic modification, transform CRC into a therapeutic response, and prevent or overcome chemoresistance.

### 2.8 Other mechanisms

Tumor metabolic abnormalities are considered the new hallmark of cancer therapy (190). Metabolic reprogramming, which involves lipid metabolism, oxidative stress, mitochondrial metabolism, and glycolysis, is significant in cancer chemoresistance (191–194). Chemoresistance mediated by tumor metabolic changes is closely related to TME and epigenetic modifications. The high expression of the methylated protein YTHDF1 promotes the high expression and activity of transglutaminase 1, resulting in CRC’s tolerance to cisplatin (177). The methylation of histone causes glutamine synthetase (GS) to be upregulated in adipocytes, and the resultant rise in the glutamine level is a potential resistance mechanism in patients with CRC to 5-FU (195). HIF-1α-induced glucose metabolism reprogramming confers 5-FU resistance to CRC cells (196). The application of drugs and small molecular compounds targeting the abnormal metabolism of tumors plays a decisive role in reversing the tolerance of traditional chemotherapy drugs and inhibiting the growth of tumors, which is the future of cancer treatment.

TCM, as an alternative and complementary medicine, has also demonstrated exceptional efficacy in regulating abnormal tumor metabolism. Moreover, natural compounds have fewer side effects while inhibiting the growth of cancer cells. By downregulating HIF-1α, Worenine and Matrine could reverse the Warburg effect and inhibit the growth of CRC cells (197, 198). Kaempferol upregulates miR-326-hnRNPA1, inhibits glycolysis mediated by PKM2, and reverses tolerance to 5-Fu in CRC (199). Jiedu Sangen decoction inhibits glycolysis in CRC by regulating the PI3K/AKT/HIF-1α signaling pathway and restoring 5-FU sensitivity (200). RA-XII is extracted from *Rubia yunnanensis* Diels and it inhibits CRC by inhibiting fatty acid synthesis (201).

The role of microbes in regulating drug metabolism is becoming increasingly clear with time. It is also established that microbes play an important role in cancer development and treatment resistance. UroA, a microbial metabolite, can inhibit ABC transporters by regulating the FOXO3-FOXM1 axis and sensitizing 5-FU-resistant CRC (202). *Lactobacillus plantarum* metabolites restored SMCT1 expression and function in 5-FU-resistant CRC cells, overcoming the double resistance of 5-FU and butyrate (203). The impairment of TGF-β signaling creates an intestinal flora imbalance and CRC resistance to 5-FU (204). Fusobacterium nucleatum promotes non-CSC lipid accumulation in CRC to achieve CSC characteristics, decreases lipid accumulation in CSC to allow it to self-renew, activates autophagy, and induces chemical resistance in CRC (205, 206). The intestinal flora can regulate the anticancer activity of oxaliplatin and cyclophosphamide and induce T-cell reaction and ROS production (207). The role of intestinal flora in chemical resistance is multifaceted, making it the guardian as well as the enemy.

Chinese herbal medicine is closely related to intestinal bacteria and plays a key role in cancer therapy. TCM influences the composition of intestinal flora, whereas intestinal microbes influence the metabolism of Chinese herbal medicines’ active compounds. This interdependent relationship offers fresh possibilities for cancer treatment. Berberine has been demonstrated to inhibit *Fusobacterium nucleatum*, regulate intestinal flora composition, relieve immunosuppression, and inhibit CRC (208, 209). Wu pill can help prevent colitis-related CRC by regulating the intestinal microbiota (210). However, ferulic acid needs to be metabolized by intestinal flora, and its product has stronger anti-cancer activity than its mother, which is used to treat chemical resistance CRC (211). Yi-Yi-Fu-Zi-Bai-Jiang-San has no direct inhibitory effect on CRC, but it significantly improves the intestinal bacteria composition and tumor immune infiltration of CRC tumor-bearing mice and inhibits the tumor growth after fecal bacteria transplantation treatment with the intestinal flora regulated by this compound (212).

## 3 Discussion

Enhancing treatment sensitivity or overcoming chemoresistance is a pressing issue in the field of cancer. Several studies have reported that TCM participates in inhibiting tumor growth and metastasis, improving tumor microenvironment, regulating cell death, inhibiting CRC progression, increasing the response of drug-resistant CRC to drug treatment, reducing toxic and side effects, improving prognosis, prolonging patient survival time (**Table 1**). TCM’s auxiliary sensitizing effect on chemotherapy sensitivity is becoming increasingly essential and evident. TCM used in combination with anticancer drugs can increase anticancer drug intracellular accumulation, prevent the original drug tolerance mechanism or mediate a new cell death mechanism, modify the tumor microenvironment, relieve the immunosuppression, erase methylation, and other reversible modifications. We thus believe that TCM has a promising future as an alternative and complementary therapy for clinical cancer treatment, whether used alone or as a sensitizer to other anticancer drugs.

**Table 1:** The main mechanisms of TCM to increase the sensitivity of chemical drugs.

Mechanisms	Chemotherapy Drugs	Type	Cell line	*In vivo or in vitro*	TCM
inhibit ABC transposters	Oxaliplatin	formula	HCT116	*in vivo*; *in vitro*	Gegen Qinlian Decoction (20)
		extract	HCT116	*in vivo*; *in vitro*	Evodiamine (29)
			SW620	*in vivo*; *in vitro*	Danshensu (140)
	5-FU	herb	HCT8	*in vitro*	*Scutellaria barbata* D. Don (22)
		extract	DLD1;HCT8; HCT116;FHC	*in vivo; in vitro*	cucurbitacin E (23)
	Paclitaxel; Adriamycin	extract	HCT8	*in vitro*	Neferine (26)
	Doxorubicin	extract	CaCo2	*in vitro*	*Arctium lappa* (27)
promote DNA damage and inhibit repair	Cisplatin	extract	HCT116	*in vivo*; *in vitro*	Shikonin (43)
		formula	HCT116; SW480	*in vitro*	Compound Kushen Injection (46)
	5-FU	extract	HCT116	*in vitro*	Curcumin (47)
promote cell death	radiation	extract	HT29	*in vivo*; *in vitro*	Curcumin (48)
	autophagy inhibitor	extract	HCT116	*in vivo*; *in vitro*	Artesunate (72)
	5-FU	extract	HCT116	*in vitro*	Curcumin (74, 75)
			HCT116; SW480	*in vivo*; *in vitro*	Andrographis (93)
		formula	HCT-8	*in vivo*; *in vitro*	Jiedu Sangen decoction (200)
	Capecitabine	extract	HCT116	*in vitro*	Curcumin (76)
	γ-radiation	extract	HCT116	*In vivo*; *in vitro*	Curcumin (77)
	Cetuximab	extract	HCT116; Lovo	*in vivo*; *in vitro*	β-elemene (95)
	anti-PD-L1 antibody	nanoparticles	HT29; Caco2; SW480	*in vivo*	Glycyrrhetinic acid (98)
inactive chemotherapy drug and change target	5-FU	extract	HCT116,	*in vitro*	Apigenin (100)
			HCT116	*in vitro*	*Coptidis Rhizoma* (119)
		formula	H63; MC38	*in vivo*; *in vitro*	Huang Qin Ge Gen Tang (116)
			LoVo	*in vitro*	Guan Chang Fu Fang (118)
Inhibit EMT	5-FU	herb	RKO; HCT15	*in vivo*; *in vitro*	*Sanguisorba officinalis* Linn (80).
		extract	HCT116	*in vitro*	Curcumin (136)
			HCT116; SW480	*in vivo*; *in vitro*	Curcumin (138)
				*in vitro*	Resveratrol (143)
			HCT116	*in vitro*	Resveratrol (144)
	Oxaliplatin	extract	HCT116; SW480	*in vivo*; *in vitro*	Cinnamaldehyde (139)
			SW620	*in vivo*; *in vitro*	Danshensu (140)
			SW480	*in vitro*	astragaloside IV (147)
Improve tumor mircoenvironment	5-FU; Oxaliplatin	extract	HCT116; HT29	*in vitro*	Curcumin (159)
	PD-1 inhibitor	formula	CT26	*in vitro*	Gegen Qinlian decoction (151) (208)
		extract	CT26	*in vivo*; *in vitro*	Andrographolide (166)
			MC38	*in vivo*	gallic acid (167)
	angiogenesis inhibitor	extract	LoVo; HCT116; HT29; SW620	*in vivo*; *in vitro*	tanshinone IIA (163)
	anti-CD47 antibody	self-assembled nanomedicine	CT26	*in vivo*	Lentinan- ursolic acid (168)
target epigenetic modification	Decitabine	extract	HCT116	*in vitro*	Curcumin (185)
	5-FU; SN38	extract	HCT116; HT29	*in vivo*; *in vitro*	Berberine (188)
	5-FU	extract	HCT8	*in vitro*	Kaempferol (199)
		formula	HCT8	*in vivo*; *in vitro*	Jiedu Sangen decoction (200)

However, the components of TCM and compound prescription are complex, and it is difficult to fully clarify its mechanism. Different from TCM, modern medicine mainly starts from the occurrence and development of cancer in the treatment of cancer, and the treatment mechanism and targeted path are clear. A variety of targeted inhibitors or small molecule drugs have been developed to overcome the chemical resistance of traditional chemotherapy drugs, such as ABC transports inhibitors, iron death inducers, autophagy inhibitors, DNA methylation inhibitors, histone deacetylase inhibitors, etc., and corresponding clinical trials have been carried out. Although these targeted preparations have shown some promising clinical results, their side effects that cannot be ignored also limit their real clinical application. ABC transports has not been approved for the treatment of malignant tumors due to its non-targeted selective inhibition and individual differences (213). The stability and dosage of epigenetic drugs also make their effects in solid tumors need to be improved (214). Under the development trend of precision medicine,TCM’s effective components have become the research focus for Chinese medicine combined with chemotherapy to reverse MDR. Presently, most TCM research on active compounds for reversing CRC MDR is mostly concentrated in flavonoids and phenols, with the greatest study in CRC in combination with traditional drugs (215). In reversing CRC multidrug resistance, Chinese medicine and its active ingredients offer both considerable potential and controversy. Different from the precise targeting characteristics of modern medicine, TCM has the characteristics of multi-component, multi-target and multi-stage in nature. For example, curcumin can inhibit DNA damage repair, inhibit EMT, down-regulate the expression and activity of HDAC and cooperate with DNA demethylation of azacytidine to reverse the resistance of decitabine and 5-FU. This feature also makes it difficult for TCM to create secondary drug resistance or even multi-drug resistance. However, because of the complex components and numerous targets of TCM and compound prescription, as well as the thinking theory and compatibility of dialectical administration in TCM theory, it is difficult to specify the exact method. Presently, the research on compound prescription is limited to a single component and mechanism and is primarily concentrated in a single extract, which does not reflect the TCM’s theory of syndrome differentiation and treatment, and the principle of compatibility among the monarch, minister assistant, and guide. Some scholars studied the optimal proportion of the main active ingredients of Aidi injection, namely cantharidin, calycosin-7-O-β-D-glucoside, ginsenoside Rc, and ginsenoside Rd, and discovered that the latter three can inhibit the degradation of cytotoxic proteins by inhibiting the activity of the ubiquitin-proteasome system, which is enhanced by cantharidin. Synergistic cantharidin can activate PP2A, activate mitochondrial apoptosis, and promote CRC cell death (189). This report fully demonstrates the scientific nature of the compatibility theory of TCM. The reasonable compatibility of TCM is also the subject of TCM modernization research. TCM research as a chemical sensitizer mainly focuses on cell and animal experiments or is based on *in vitro* research. Although several clinical trials of TCM for CRC have been conducted, there is still a lack of evident clinical randomized controlled trials, and it has not yet become the first line of clinical medication. Weak absorption, low solubility, bioavailability, and unknown side effects have always limited the development of TCM’s global expansion. However, with the development of nanotechnologies, such as liposomes, nanoparticles, and bionic drug delivery systems, the abovementioned defects of active ingredients of TCM have gradually been addressed (216–219). It is critical to accelerate the transformation of TCM experimental research findings into clinical applications by conducting evidence-based medicine and component research to assess efficacy and safety. Currently, the establishment of the State Key Laboratory of Traditional Chinese Medicine Components and its component bank and the Food and Drug Administration’s approval of clinical TCM research have all accelerated the pace of modern TCM research and development.

## 4 Conclusion and perspective

With an increasing incidence rate of CRC morbidity and mortality, the concerns related to CRC treatment include improving drug sensitivity and reversing chemoresistance. TCM can work in conjunction with anticancer medications to lessen side effects and boost effectiveness, boost intracellular anticancer drug accumulation, block initial drug tolerance, mediate cell death, enhance tumor microenvironment, relieve immunosuppression, erase methylation and other reversible modifications, and produce significant anticancer efficacy. TCM has shown great advantages in the process of increasing chemotherapy sensitivity with its multi-component, multi-target and multi-stage treatment characteristics different from modern medicine. We believe TCM has a promising future as an alternative and complementary therapy for clinical CRC treatment in developing new sensitizers to other anticancer drugs.

## Author contributions

XL and XH contributed into the writing and revision of the manuscript. XY, YY and HZ draw figures and tables. All authors contributed to the article and approved the submitted version.
